# Russian isolates enlarge the known geographic diversity of *Francisella tularensis* subsp. *mediasiatica*

**DOI:** 10.1371/journal.pone.0183714

**Published:** 2017-09-05

**Authors:** Vitalii Timofeev, Irina Bakhteeva, Galina Titareva, Pavel Kopylov, David Christiany, Alexander Mokrievich, Ivan Dyatlov, Gilles Vergnaud

**Affiliations:** 1 State Research Center for Applied Microbiology and Biotechnology (SRCAMB), Obolensk, Moscow Region, Russia; 2 Institute for Integrative Biology of the Cell (I2BC), CEA, CNRS, Univ. Paris-Sud, Université Paris-Saclay, Gif-sur-Yvette cedex, France; Midwestern University, UNITED STATES

## Abstract

*Francisella tularensis*, a small Gram-negative bacterium, is capable of infecting a wide range of animals, including humans, and causes a plague-like disease called tularemia—a highly contagious disease with a high mortality rate. Because of these characteristics, *F*. *tularensis* is considered a potential agent of biological terrorism. Currently, *F*. *tularensis* is divided into four subspecies, which differ in their virulence and geographic distribution. Two of them, subsp. *tularensis* (primarily found in North America) and subsp. *holarctica* (widespread across the Northern Hemisphere), are responsible for tularemia in humans. Subsp. *novicida* is almost avirulent in humans. The fourth subspecies, subsp. *mediasiatica*, is the least studied because of its limited distribution and impact in human health. It is found only in sparsely populated regions of Central Asia. In this report, we describe the first focus of naturally circulating *F*. *tularensis* subsp. *mediasiatica* in Russia. We isolated and characterized 18 strains of this subspecies in the Altai region. All strains were highly virulent in mice. The virulence of subsp. *mediasiatica* in a vaccinated mouse model is intermediate between that of subsp. *tularensis* and subsp. *holarctica*. Based on a multiple-locus variable number tandem repeat analysis (MLVA), we show that the Altaic population of *F*. *tularensis* subsp. *mediasiatica* is genetically distinct from the classical Central Asian population, and probably is endemic to Southern Siberia. We propose to subdivide the *mediasiatica* subspecies into three phylogeographic groups, M.I, M.II and M.III.

## Introduction

*F*. *tularensis* is a small Gram-negative aerobic coccobacillus. It is a facultative intracellular parasite capable of infecting a wide range of animals and causing a plague-like disease called tularemia. People, particularly those living within endemic foci and working as farmers, hunters, foresters, or meat processing and leather industry workers, are at risk of tularemia infection. This bacterium can be acquired in a variety of ways, including through bites from arthropods, inhalation, direct contact, or ingestion of contaminated animal tissue (often rabbits and rodents), or contact with contaminated soil or water [1–6].

Currently *F*. *tularensis* is divided into four subspecies: *tularensis* (*nearctica*), *holarctica* (*palaearctica*), *mediasiatica*, and *novicida*, which differ in their distribution and virulence in humans [2, 7, 8]. *Tularensis* and *holarctica* are the most common and well-described subspecies. Subspecies *tularensis* is mainly found in North America, and shows the greatest virulence in humans [8, 9]. The mortality rate in humans can reach 24% in untreated patients [10]. Subspecies *holarctica* is commonly found in America and Eurasia, i.e., throughout the Northern Hemisphere. It is less virulent for humans than subsp. *tularensis*, with a case fatality rate of up to 7% in untreated patients [9].

*F*. *tularensis* subsp. *novicida* is present in North America, but some strains have been isolated in tropical Australia and Southeast Asia (clinical isolates only) [11, 12]. To date, the status of *novicida*, separate species or subspecies, remains non-standardized and controversial [4, 13–16]. Although this subspecies is almost avirulent in humans, there have been several reports of this subspecies isolated from patients [11, 12, 17–19].

*F*. *tularensis* subsp. *mediasiatica* remains the least studied and understood subspecies. It was found in Central Asia [20, 21], in some regions of Kazakhstan and Turkmenistan. This geographic location is responsible for the subspecies name—in Russian “Middle Asia” is a synonym for “Central Asia”. Nothing is known about the potential for this subspecies to cause disease in humans [22, 23]. The absence of clinical cases is indirect evidence suggesting that virulence in humans is low at best. Virulence in hares is similar to virulence of subsp. *holarctica* (reviewed in [2]).

In 2013, *F*. *tularensis* subsp. *mediasiatica* was reported to be present in Russia [24]. Three of four Altai-originating strains (designated in this article as A554, A678, and A823) were shown to belong to subsp. *mediasiatica*. For description of these isolates we used Multiple-locus VNTR (variable number of tandem repeat) analysis (MLVA). This approach has been previously shown to be very efficient as a first line assay able) to distinguish among even very close genetic relatives, including individual strains [2]. MLVA profiles of these strains were similar, and they differed slightly from MLVA-profiles of previously reported Central Asian strains.

In 2013–2014, we received 15 additional strains of *F*. *tularensis* from an Altai anti-plague station and Federal Healthcare Service Center for Hygiene and Epidemiology in the Altai region. In the present report, we compare the MLVA and biochemical data from these strains with data from the rest of our collection and from the literature. We show that they all belong to subsp. *mediasiatica*, but confirm that they constitute a distinct cluster, providing new insight on the intra-subspecies structure. In addition, we investigated the virulence of Altai strains in laboratory mice.

## Materials and methods

### Strains

Strains of *F*. *tularensis* used in this study are listed in **S1 Table**. Eighteen strains were isolated within the Republic of Altai and Altai Territory in 2011–2014 by the Federal Healthcare Service Center for Hygiene and Epidemiology in the Altai region and by the Altai anti-plague station. All Altai strains were isolated from ticks and dead rodents, none were clinical isolates.

### Bacterial culture

Strains were grown at 37°C on solid (FT-agar) and liquid (FT-broth) nutrient media (SRCAMB, Obolensk, Russia). The composition of the FT-agar was 3.8% erythritol-agar, 1% dried bovine blood, 1% glucose, 0.05% cysteine, and 0.0025% thiamine chloride at pH 7.2. The composition of the FT-broth was 2% casein enzymatic hydrolysate, 1% yeast extract, 1.2% KH_2_PO_4_, 1% glucose, 0.001% cysteine, and 0.001% FeCl_2_ at pH 7.2.

### Polymerase chain reaction (PCR)

PCR was performed using a Mastercycler Gradient thermal cycler (Eppendorf, Hamburg, Germany). Each reaction mix contained 5 μl of 5×qPCRmix-HS (Evrogen, Moscow, Russia). This mix corresponds to a final concentration of 200 μM for each dNTP and 3 mM MgCl_2_ with one unit of Taq DNA polymerase and 10 to 50 ng of DNA per 25 μl reaction. Primers (Syntol, Moscow, Russia) were added at a final concentration of 10pM.

### Multiple-locus VNTR analysis (MLVA) genotyping

We used 15 among the 25 VNTR loci described in [25]: FtM3, FtM5, FtM6, FtM7, FtM8, FtM9, FtM10, FtM11, FtM12, FtM13, FtM16, FtM19, FtM20, FtM23, FtM24. *In vitro* MLVA genotyping was done as previously described [25] except for the use of regular, non-fluorescent primers, agarose gels and monoplex PCR. PCR products size was evaluated using agarose gel-electrophoresis. The PCR products and a 20 bp ladder (Bio-Rad, USA) were electrophoresed at 100 V for 240 min on a 32-cm length 3% agarose gel prepared in 0.5× TBE. The DNA fragments were visualized with ethidium bromide staining and ultraviolet (254 nm) using the Doc-Print gel documenting system and PhotoCaptMw software version 99.04 (Vilber Lourmat, Marne-la-Vallée, France). PCR products larger than 600 bp were reanalyzed on 2% agarose gel for better resolution. Also in these few cases we confirmed the size of amplicon using Experion™ Automated Electrophoresis System (BioRad, Hercules, USA) and by sequencing the fragment, followed by a direct count of the number of repeats.

Cluster analyses were run using BioNumerics version 7.6.2 (Applied-Maths, Belgium).

### Single-primer genotyping

Single-primer genotyping was performed by PCR with primer Chif1 (5′-CTAGGGCTGGTGGG-3′). Thermocycling parameters were the following: 94°C for 3for3 min; 30 cycles of 94°C for 30 sec, 54°C for 30 sec, and 72°C for 1 min; and then a final elongation at 72°C for 5 min. The PCR products and a 100 bp ladder (Bio-Rad, USA) were electrophoresed at 100 V for 90 min on a 1% agarose gel prepared in 1× TBE and visualized with ethidium bromide staining and ultraviolet (254 nm) using the Doc-Print gel documenting system (VilberLourmat, France). The particular subspecies was determined by the size of the obtained amplicons (**Table 1**).

**Table 1:** Presence of diagnostically important amplicons in the single-primer typing system for different subspecies of *F*. *tularensis*.

approximate size of amplicon, bp	subspecies			
	*holarctica*	*tularensis*	*mediasiatica*	*novicida*
1460*	**+**	**+/-**	**+/-**	**+**
**960**	**-**	**+**	**+**	**-**
**560**	**+**	**-**	**-**	**-**
**530**	**-**	**+**	**-**	**-**
**390**	**-**	**+**	**+**	**-**
**290**	**+**	**+**	**+**	**+**

* 1460 bp amplicon is present in all subspecies, but in lower amount in subsp *tularensis* and *mediasiatica* strains (designated as+/-)

### *In silico* MicrobesGenotyping database

The MLVA data described in this report is included in S1 Table and has been imported into the public *Francisella* database at http://microbesgenotyping.i2bc.paris-saclay.fr/.

### Draft whole genome sequencing and whole genome single nucleotide polymorphism (SNP) analysis

Complete genomes and genome assemblies were downloaded via Genbank/NCBI. Sequence Reads Archives (SRA) files were downloaded from the European Nucleotide Archive (ENA). SRA files quality was checked using FastQC. Reads with poor quality towards the ends were trimmed using Fastx_trimmer from the FASTX-Toolkit. Complete genomes and assemblies were converted into artificial 300 bp long reads using wgsim without introducing errors or mutations. Read files were then imported into BioNumerics version 7.6.2 to be mapped on the reference genome. SNPs were called using the BioNumerics SNP analysis pipeline with the “strict SNP clustering (closed SNP dataset)” option.

### Biochemical properties determination

#### Fermentation of glycerol

Acid production during glycerol fermentation was estimated in an FT-broth without glucose or casein hydrolysate, but with the addition of 0.2% glycerol (w/v) and 0.02%(w/v) phenol red as an indicator.

#### Detection of citrulline ureidase activity

We determined the activity of *F*. *tularensis* using a color test with ninhydrin [26] based on ability of citrulline ureidase to degrade citrulline to ornithine, which, on reaction with ninhydrin reagent gives a pink coloration [27].

A 1.0-ml sample of bacterial suspension (10^10^ bacteria) in 0.1 M phosphate buffered saline (PBS, pH 6.5) was mixed with 1.0 ml of 0.7% (w/v) l-citrulline (Sigma Chemical Co., St. Louis, MO, USA) and incubated for 20 h at 30°C. An aliquot (0.01 ml) of the mixture was removed and added to 0.49 ml of distilled water, 1.0 ml of freshly prepared ninhydrin reagent (625 mg of ninhydrin [Sigma] in 10 ml of 6 M H_3_PO_4_ and 15 ml of glacial acetic acid), and 1.5 ml of acetic acid. Samples were boiled for 1 h, and pink colored samples were considered to have citrulline ureidase activity.

#### Beta-lactamase activity

One microbiological wire loop of each tested strain was suspended into 1 ml of phosphate-buffered saline (PBS). One hundred μl of this suspension were mixed with 10 μl of nitrocefin solution (500 mg/ml).

After incubation at room temperature during one hour, red colored samples were considered to have beta-lactamase activity [28].

### Animal experiments

#### Ethics statement

All protocols for animal experiments were approved by the State Research Center for Applied Microbiology and Biotechnology Bioethics Committee (Permit No: VP-2016/2). They were performed in compliance with the NIH Animal Welfare Insurance #A5476-01 issued on 02/07/2007 and the European Union guidelines and regulations on handling, care, and protection of laboratory animals (http://ec.europa.eu/environment/chemicals/lab_animals/home_en.htm).

#### Mice

Five-to-eight-weeks-old BALB/C mice (not-specific pathogen free) of both genders, weighing 18–20g (purchased from Laboratory Animals Breeding Center, Shemyakin and Ovchinnikov Institute of Bioorganic Chemistry, Russia), were used in experiments. The mice were housed in polycarbonate cages with space for comfortable movement and easy access to food and water, under constant temperature and humidity conditions (22°C ± 2°C and 50% ± 10%, respectively) and a 12-hour light/12-hour dark cycle. Mice were fed Mouse Mixed Fodder PK-120 (Laboratorkorm, Russia) and provided tap water *ad libitum* throughout the study.

A minimum number of mice was used for experiments. The mice were randomly divided into experimental groups.

Approved protocols provided scientifically validated humane endpoints, including pre-set criteria for euthanasia of moribund mice by CO_2_ inhalation. In our animal studies, mice were euthanized when they became lethargic, dehydrated, moribund, unable to rise, or non-responsive to touch.

The health condition of the animals was monitored at least twice a day.

#### Virulence determination

Mice were inoculated subcutaneously with 0.1 ml *F*. *tularensis* cells in PBS (5×10^1^ to 6.25×10^2^ colony forming units (CFU) per animal) in the inner part of the upper thigh.

To determine the actual infectious dose, serial ten-fold dilutions of bacterial cells were plated onto FT-agar, and the colonies were counted after incubation at 37°C for 48 hours. Dead mice were autopsied and subjected to bacteriological studies. The surviving animals were monitored for 14 days.

#### Vaccination and challenge

BALB/C mice were inoculated subcutaneously with live vaccine strain 15 NIIEG (20 CFU per mouse in 0.1 ml of PBS) in the inner part of the upper thigh. Three weeks later, the mice were challenged with virulent strains of *F*. *tularensis* (1000 CFU per mouse). Survival and body weights of immunized mice were monitored every day for 14 days. Mice were weighed in groups of six animals (or less in the event of a death) in a special tray, and the average weight was calculated.

Percent body weight loss was calculated as follows: 100 − final weight/initial weight ×100.

#### Statistics

Survival curves were generated by plotting the proportion of surviving mice against the time of death (days). For comparison of mouse weights, data was compiled from four independent experiments. The mean ± standard deviation (SD) was calculated for the percentage weight loss and mean time to death. A significant difference in weight loss or time to death between groups of animals was determined using a Student’s *t*-test with significance set at p<0.05.

### Map-containing figures

Map-containing figures were designed using Google-maps service (map data 2017). Maps are the property of Google and partners.

## Results

### MLVA genotyping of a Russian collection of *F*. *tularensis*

Twenty-five polymorphic tandem repeats have been described by Johansson et al. [25]. Different subsets have been subsequently selected to suit specific purposes. We retained the 15 loci with repeat size of 9 bp or more which can be typed confidently, not only on relatively expensive capillary electrophoresis equipment, but also on regular agarose gels (FtM3, FtM5, FtM6, FtM7, FtM8, FtM9, FtM10, FtM11, FtM12, FtM13, FtM16, FtM19, FtM20, FtM23, FtM24). The example of locus size estimating is in S1 Appendix. Low cost and robustness is one major advantage of MLVA as first-line assay and quality check of strain identity prior to running e.g. whole genome sequencing. This is essential for rapid epidemiological investigation and local investigations by regional anti-plague and medical institutions. We first evaluated the validity of this assay by using the MLVA data associated with [25] and accessible via http://microbesgenotyping.i2bc.paris-saclay.fr/. Using all 25 loci, Johansson et al. resolved 119 genotypes among 192 strains. Subspecies were clustered as expected, and additional subdivisions were proposed, *tularensis* A.I and A.II, *holarctica* B.I to B.V. In comparison MLVA15 resolves 104 genotypes. The previously defined groups are conserved, except for B.I, B.II and B.III which are not resolved when using this subset (analysis not shown).

Since 2008 SRCAMB acts as the Tularemia Reference Center and has assembled a collection of 173 *F*. *tularensis* live strains or DNA samples collected by Russian anti-plague and medical institutions (**S1 Table**). The strains were genotyped using MLVA15. One hundred and twelve genotypes are resolved. The Simpson’s diversity index was 0.9894 (95% confidence interval 0.9850–0.9938). The number of detected alleles and Simpson’s diversity index calculated at all used VNTR markers are indicated in **Table 2.** In agreement with previous reports, VNTR locus FtM3 is the most variable locus.

**Table 2:** Number of detected alleles and Simpson’s diversity index calculated at 15 VNTR markers using the SRCAMB collection of 173 strains.

Locus	Number of alleles	Simpson’s Index (confidence interval)
FtM3	37	0.95063(0.9407–0.9606)
FtM5	8	0.29074(0.2020–0.3795)
FtM6	7	0.67039(0.6169–0.7239)
FtM7	8	0.28224(0.1934–0.3710)
FtM8	5	0.38855(0.3057–0.4714)
FtM9	4	0.15308(0.0795–0.2266)
FM10	9	0.14384(0.0710–0.2167)
FM11	6	0.27613(0.1915–0.3607)
FM12	3	0.32795(0.2472–0.4087)
FM13	2	0.09920(0.0383–0.1601)
FM16	2	0.05614(0.0084–0.1039)
FtM19	4	0.34576(0.2689–0.4226)
FtM20	14	0.44475(0.3505–0.5390)
FtM23	2	0.02286(0.0000–0.0544)
FtM24	2	0.40695(0.3474–0.4665)

**Fig 1:**
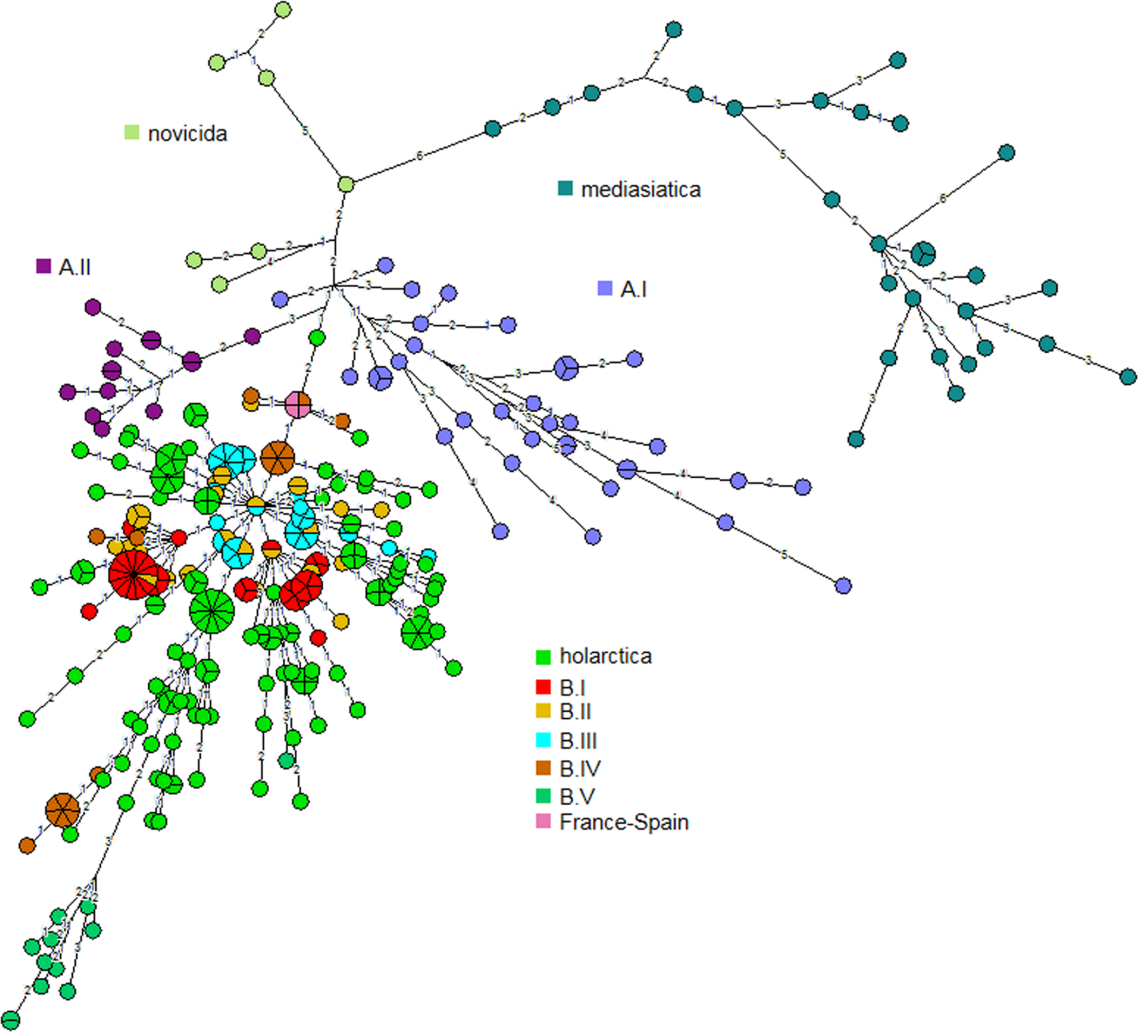
Minimum spanning tree deduced from MLVA15 data for 365 entries (192 from [25] and 173 from the present investigation). Branches with length up to four are drawn. Short unlabeled branches correspond to a distance of one locus difference. Branch length of five and more are not drawn.

The data was merged with MLVA15 data from Johansson et al., to produce a minimum spanning tree. **Fig 1**shows the resulting clustering, the color code reflects subspecies and additional subdivisions. The main cluster corresponds to *holarctica*. Subspecies *tularensis* subgroups A.I and A.II are clearly identified. All strains of Russian geographic origin are *holarctica*, with the exception of *mediasiatica* strains. The *mediasiatica* group is the most distantly related, with a long branch (six differences) connecting it to the *novicida* subspecies. Within *mediasiatica*, two additional long branches are detected.

Although the origin of the Altai strains is not typical for subsp. *mediasiatica*, clusterisation together with «classical» *mediasiatica* strains points that these strains do belong to subsp. *mediasiatica*. Previously, *F*. *tularensis* subsp. *mediasiatica* was believed to be abundant only in Central Asia. Until 2011, there have been no strains isolated in the territory of Russia, other than those belonging to subsp. *holarctica*. The presence of subsp. *mediasiatica* strains in Russia can be explained by two alternative hypotheses, recent accidental dissemination beyond its native area, or underestimation of the distribution of this subspecies.

**Fig 2:**
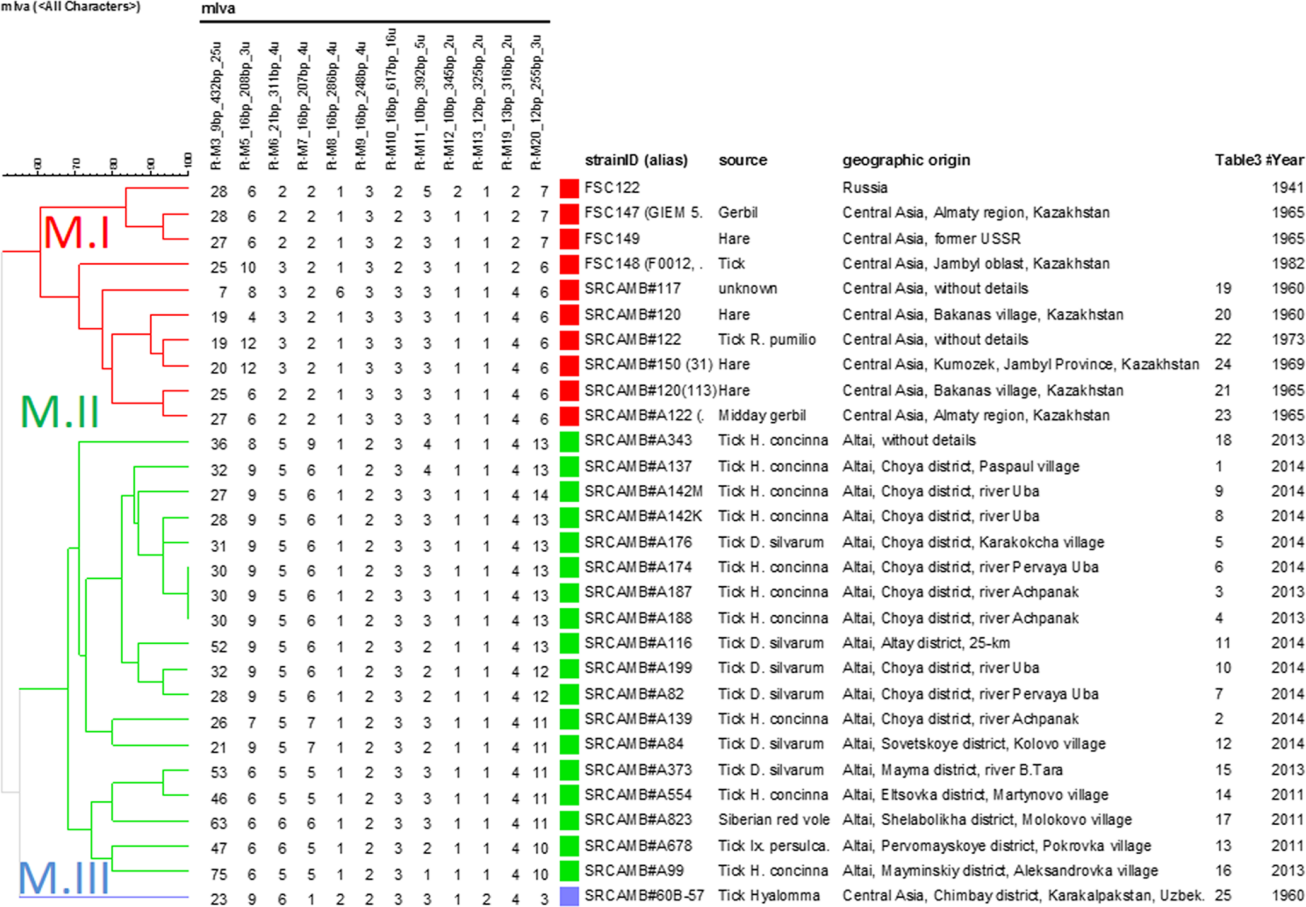
UPGMA clustering analysis of mediasiatica strains based on MLVA15 data. The dendrogram was constructed using UPGMA (Unweighted Pair Group Method with Arithmetic Mean). The MLVA data of 12 loci are shown (loci Ft-M16, Ft-M23 and Ft-M24 with an identical allele 1 in all strains are masked). The Table 3# index refers to the location indicated in **Fig 4**and numbering in **Table 3**. The cluster cut-off value of 60% defines three groups, labelled M.I (Central Asian origin, red), M.II (Altaic origin, green) and M.III (Karakalpak, blue). Strains from SRCAMB collection are indicated using prefix SRCAMB#.

**Fig 3:**
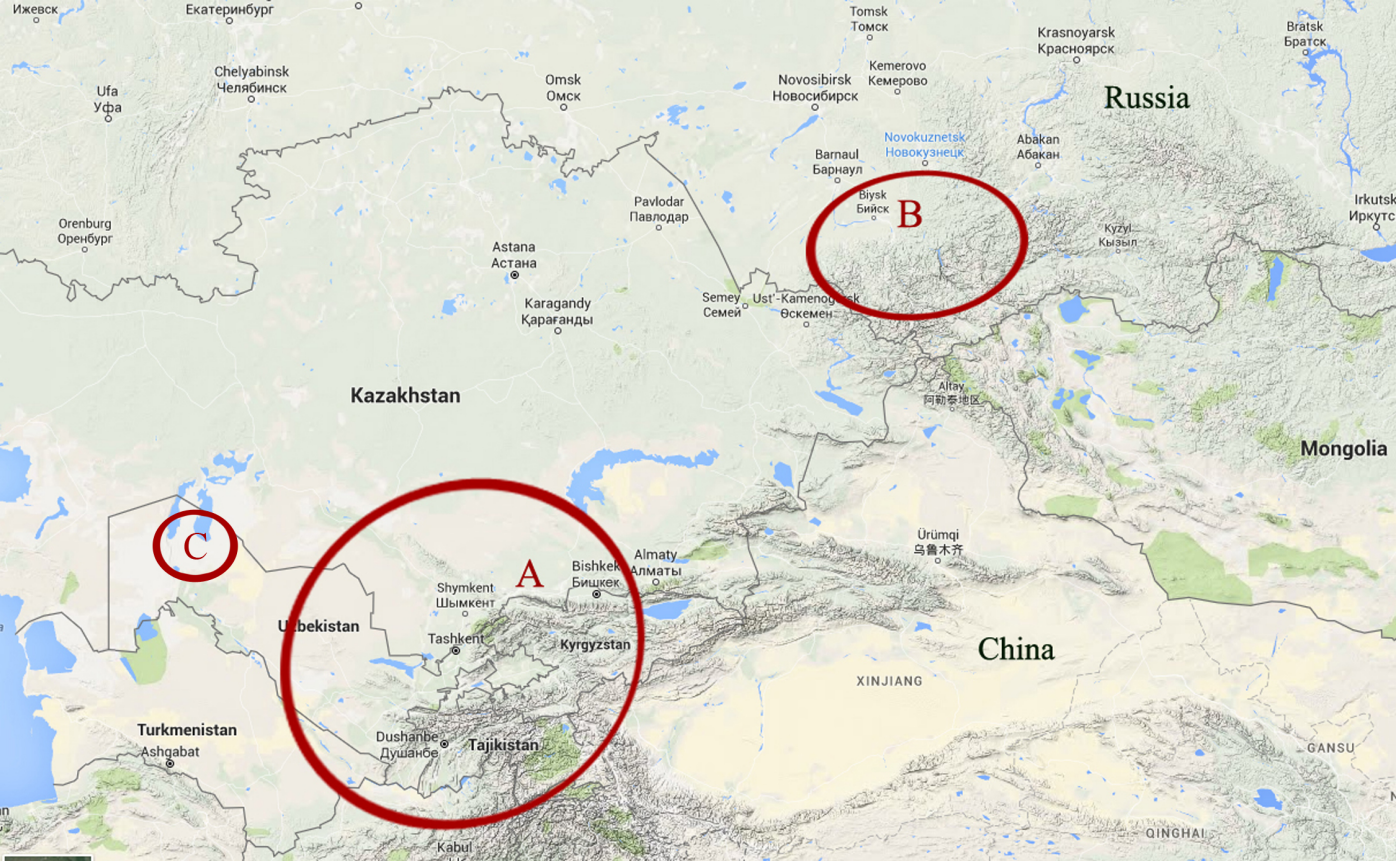
Distribution of *F*. *tularensis* subsp. *mediasiatica*. A, Central Asia region; B, Altai region; C, Karakalpakstan.

**Fig 4:**
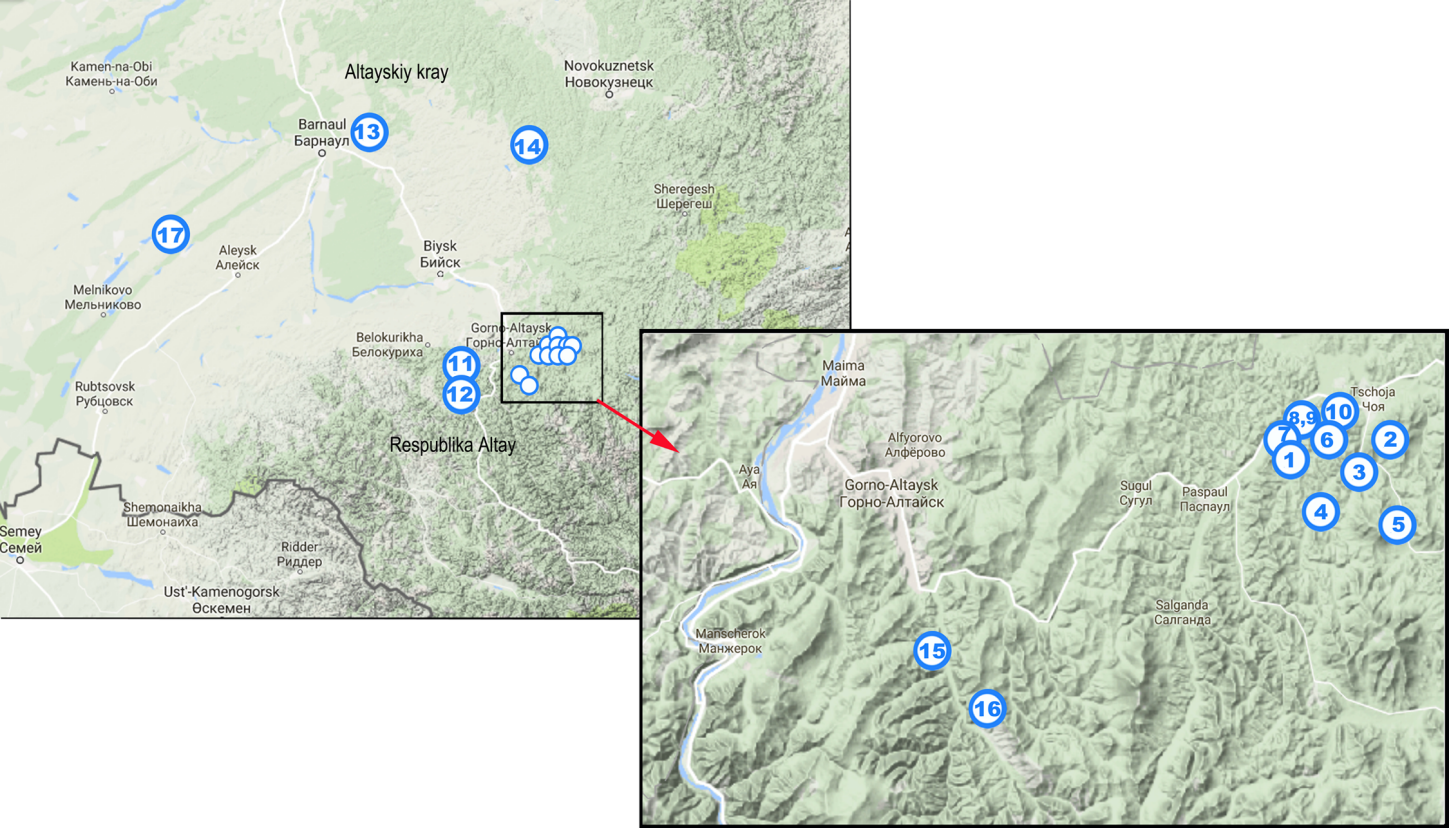
Isolation points of Altaic *mediasiatica* strains. Strains are designated by numbers: 1—A137; 2—A139; 3—A187; 4—A188; 5—A176; 6—A174; 7—A82; 8—A142 K; 9-A142 M; 10—A199; 11—A116; 12—A84; 13—A678; 14—A554; 15—A373; 16—A99; and 17—A823 as also shown in **Table 3**and **Fig 2**.

**Fig 2**presents a focus on the twenty-nine mediasiatica strains including four from the Johansson et al. investigation. The figure shows available metadata and MLVA genotypes. A first subcluster subsequently called M.I in keeping with previous nomenclature contains the Johansson et al. strains and six additional strains isolated in Central Asia, mostly Kazakhstan (region A **Fig 3**). A second subcluster subsequently called M.II comprises the 18 strains isolated within the Republic of Altai and Altai Territory in 2011–2014 (region B **Fig 3**and **Fig 4**). A single strain isolated in Karakalpakstan (region C **Fig 3**) forms a separate subcluster M.III. The position of the three subclusters is also shown in **Fig 1**. **Table 3**provides the list of 25 mediasiatica strains investigated here, together with available metadata. **Table 4**illustrates some of the major differences in terms of MLVA genotypes between M.I, M.II and M.III. M.II strains of Altaic origin were characterized by relatively large alleles at loci Ft-M3, Ft-M6, Ft-M7, and Ft-M20.

**Table 3:** The list of 25 *mediasiatica* strains investigated in this report.

№	Strain	Region	Geographic Origin	Source	Year
1	A137	Altai	Paspaul village, Choya district, Altai	Tick H. concinna	2014
2	A139	Altai	Choya district, river Achpanak, Altai	Tick H. concinna	2014
3	A187	Altai	Choya district, river Achpanak, Altai	Tick H. concinna	2013
4	A188	Altai	Choya district, river Achpanak, Altai	Tick H. concinna	2013
5	A176	Altai	Karakokcha village, Choya district, Altai	Tick D. Silvarum	2014
6	A174	Altai	Choya district river Pervaya Uba, Altai	Tick H. concinna	2014
7	A82	Altai	Choya district, river Pervaya Uba, Altai	Tick D. silvarum	2014
8	A142 K	Altai	Choya district river Uba, Altai	Tick H. concinna	2014
9	A142 M	Altai	Choya district river Uba, Altai	Tick H. concinna	2014
10	A199	Altai	Choya district, river Uba, Altai	Tick D. Silvarum	2014
11	A116	Altai	Altay district, 25-km, Altai	Tick D. silvarum	2014
12	A84	Altai	Kolovo village, Sovetskoye district, Altai	Tick D. silvarum	2014
13	A678	Altai	Pokrovka village, Pervomayskoye district, Altai	Tick Ix. persulcatus	2011
14	A554	Altai	Martynovo village, Eltsovka district, Altai	Tick H. concinna	2011
15	A373	Altai	Mayma district, river B.Tara, Altai	Tick D. silvarum	2013
16	A99	Altai	Aleksandrovka village, Mayminskiy district, Altai	Tick H. concinni	2013
17	A823	Altai	Molokovo village, Shelabolikha district, Altai	Siberian red vole	2011
18	A343	Altai	Altai, without details	Tick H. concinna	2013
19	117	Central Asia	unknown	unknown	1960
20	120	Central Asia	Bakanas village, Kazakhstan, Central Asia	Hare	1960
21	120 (113)	Central Asia	Bakanas village, Kazakhstan, Central Asia	Hare	1965
22	122	Central Asia	Central Asia, without details	Tick R. pumilio	1973
23	A122 (543)	Central Asia	Almaty region, Kazakhstan, Central Asia	midday gerbil	1965
24	150 (31)	Central Asia	Kumozek, Jambyl Province, Kazakhstan, Central Asia	Hare	1969
25	60B-57	Central Asia, Karakalpak	Chimbay district, Karakalpakstan, Uzbekistan, Central Asia	Tick Hyalomma	1960

**Table 4:** Remarkable differences of Tandem repeat copy number between the Central Asian M.I, Altaic M.II and Karakalpak M.III strains.

Locus	Region of origin		
	M.I Central Asia *n = 6+4*	M.II Altai *n = 18*	M.III Karakalpakstan (Central Asia) *n = 1*
Ft-M3	7–27	21–75	23
Ft-M6	2–3	5–6	3
Ft-M7	2	5–9	1
Ft-M20	6	10–14	3

For instance thirteen of eighteen M.II strains of Altai origin showed more than 30 repeats at locus Ft-M3, whereas M.I Central Asian strains showed less than 27.

The behavior of the atypical strain, 60B-57, is reminiscent of the Central Asian strains. Interestingly strain 60B-57appears to define a third group (**Fig 2**, M.III label). This strain is the only strain originating from Karakalpakstan in Western Uzbekistan further illustrating the relation between MLVA genotypes and geographic origin (**Fig 3**). This strong anchorage of *mediasiatica* with geography is suggesting that there is limited spreading between different ecological niches and consequently that the diversity of *mediasiatica* may be underestimated, given its low impact on human health.

### Establishing simple first-line assays for subpopulation assignments

#### MLVA9_Obolensk_, as a first line MLVA typing assay

In order to simplify the initial genotyping steps and lower the analysis cost, we have evaluated a minimum panel of VNTRs able to efficiently resolve *F*. *tularensis* strains naturally present in Russia and Central Asia. Using the MLVA data from the 144 corresponding strains, the Automated Selection of Typing Target Subsets (AuSeTTS) software [29] provides a list of nine VNTR loci sufficient to achieve the same discrimination as the 15 loci in the tested data set: Ft-M3, Ft-M5, Ft-M6, Ft-M7, Ft-M8, Ft-M12, Ft-M19, Ft-M20, Ft-M24. In keeping with previous similar MLVA assays, and in order to distinguish this set from other selections with an identical number of loci, we call this selection MLVA9_Obolensk_. An even simpler MLVA6 assay was proposed by Gürcan et al. [30]. Four of the six loci proposed by these authors are included in the MLVA9_Obolensk_ panel (Ft-M3, Ft-M6, Ft-M20, Ft-M24), the last two (Ft-M21 and Ft-M22) with seven and six base-pair repeat units respectively, were not included in the present investigation. Similarly, Vogler et al. [31] proposed an MLVA10 assay, comprising Ft-M2, Ft-M3, Ft-M4, Ft-M5, Ft-M6, Ft-M10, Ft-M20, Ft-M22, Ft-M23, Ft-M24. Table 5 shows the composition of the three MLVA assays. The three assays can be compared using the Johansson et al. investigation **[25]**, which constitutes the most complete dataset. Whereas 119 genotypes are resolved among the 192 strains when using all 25 loci, MLVA6, MLVA9_Obolensk_ and MLVA10 resolve 111, 101 and 116 genotypes, respectively.

**Table 5:** Composition of proposed selections of VNTR loci for simplified MLVA assays and comparison from *in silico* derived data.

Locus					MLVA assay		
	Repeat unit size	Allele number	HGDI^1^	Standard deviation	MLVA9_ObObolensk_	MLVA6 [30]	MLVA10 [31]
Ft-M2	6bp	2020	0.45294529	[0.3640,0.5419]			X
Ft-M3	9bp	3030	0.94629462	[0.9358,0.9566]	X	X	X
Ft-M4	5bp	66	0.58375837	[0.5283,0.6391]			X
Ft-M5	16bp	66	0.21352135	[0.1350,0.2920]	X		X
Ft-M6	21bp	66	0.64616461	[0.5985,0.6936]	X	X	X
Ft-M7	16bp	66	0.19481948	[0.1189,0.2707]	X		
Ft-M8	16bp	66	0.31633163	[0.2307,0.4019]	X		
Ft-M10	16bp	1515	0.44634463	[0.3569,0.5356]			X
Ft-M12	10bp	2	0.03090309	[0.0000,0.0656]	X		
Ft-M19	13bp	2	0.40184018	[0.3440,0.4596]	X		
Ft-M20	12bp	1515	0.34443444	[0.2558,0.4330]	X	X	X
Ft-M21	7bp	66	0.35323532	[0.2764,0.4300]		X	
Ft-M22	6bp	55	0.642642	[0.6115,0.6725]		X	X
Ft-M23	23bp	33	0.17171717	[0.1025,0.2410]			X
Ft-M24	21bp	33	0.49934993	[0.4780,0.5206]	X	X	X

^1^ the Hunter-Gaston diversity index was calculated using the Johansson et al, **[25]** dataset for 192 strains.

#### Determination of subspecies by single primer amplification

**Fig 5:**
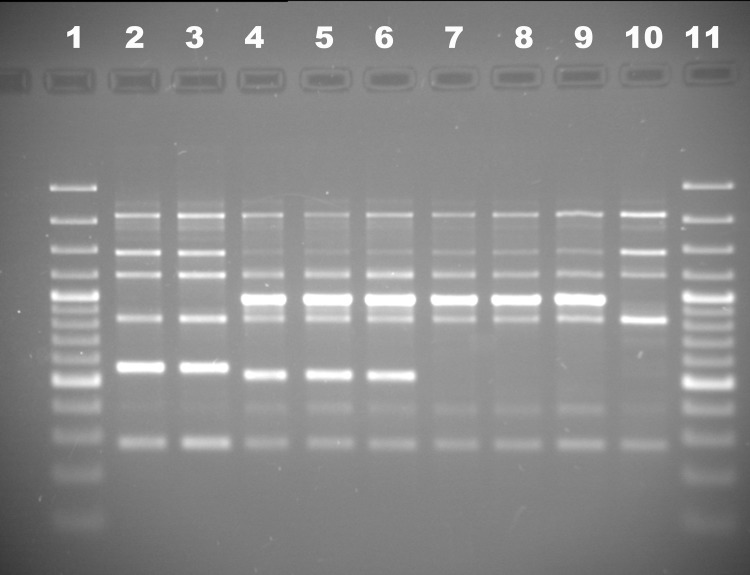
Single primer—genotyping of representative *F*. *tularensis* strains. The figure shows the distribution of the single-primer PCR amplicons for representatives from all four subspecies. Lines 1, 11– GeneRuler^TM^ 100 bp Plus DNA Ladder; 2– subsp. *holarctica* 503; 3– subsp. *holarctica* А1045; 4– subsp. *tularensis* SCHU S4; 5– subsp. subsp. *tularensis* BB-399; 6– subsp. *tularensis* 8859; 7– subsp. *mediasiatica* 120; 8 –subsp. *mediasiatica* A678; 9– subsp. *mediasiatica* A554; 10– subsp. *novicida* 112.

We previously developed a *F*. *tularensis* typing assay using a single-primer PCR (**Fig 5**). This method which is essentially a Random Amplified Polymorphic DNA (RAPD) assay [32] is used for the rapid determination of the subspecies of strains arriving in the SCRAMB collection. We observed that this method is able to distinguish all four subspecies by analysing the PCR product using agarose gel electrophoresis. **Fig 5**shows the results obtained for nine representative strains, including two *holarctica*, three *tularensis*, three *mediasiatica*, and one *novicida*.

All *mediasiatica* strains show an identical pattern. **Fig 5**shows the result obtained with M.I strain 120 and M.II strains A678 and A554. Therefore this simple assay appears to be valid also for the new *mediasiatica* strains.

### Phylogeny by whole genome SNP analysis of *F*. *tularensis* including one *mediasiatica* M.II representative

**Fig 6:**
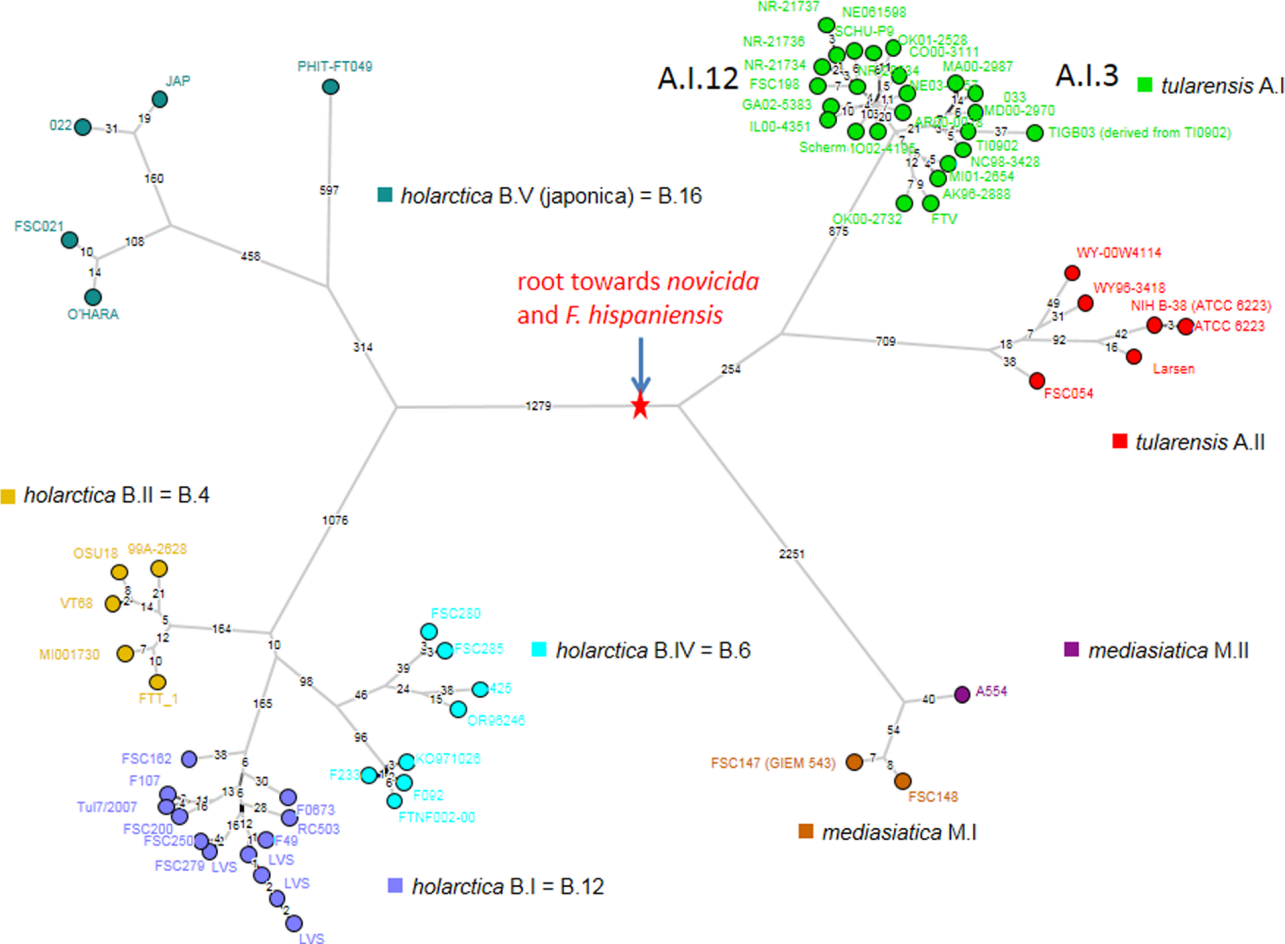
Minimum spanning tree deduced from whole genome SNP analysis. The 9,737 SNPs constitute a tree with a size of 9,819 SNPs corresponding to an homoplasy level of 0.8%. The red star indicates the position of the MRCA. The tree includes a few laboratory-derived strains: four LVS representatives within holarctica B.I, two ATCC 6223 representatives within tularensis A.II and at least five SCHU S4 variants among A.I. The star-like pattern around the SCHU S4 and short radiating branches may be the result of a very recent outbreak. The correspondence between lineage names defined by MLVA and the more recent names defined by SNPs is indicated, as in B.I = B.12.

In order to confirm the clustering suggested by MLVA analysis, we sequenced one representative strain from the *mediasiatica* M.II group, strain A554. Additional public whole genome sequence data including full genomes, contigs, scaffold and reads was downloaded from the European Nucleotide Archive (ENA). Data from 65 strains was retained after removal of datasets of insufficient quality, or representing variants of laboratory strains or duplicates. This final set includes 31 *holarctica*, 31 *tularensis* and three *mediasiatica* strains (**S2 Table**). The *mediasiatica* strains are A554 from M.II (this report) and FSC147 (alias GIEM 543) and FSC148 belonging to M.I, both from Kazakhstan. A total of 9,737 core genome SNPs is retained by the SNPs calling pipeline. The resulting minimum spanning tree has a size of 9,819 indicating a homoplasy of 0.8This observation is in agreement with previous analyses indicating that evolution within *Francisella tularensis* stricto sensu (not including *F*. *novicida*) is clonal [33, 34]. **Fig 6**shows the minimum spanning tree of the three clonal subspecies. The Most Recent Common Ancestor (MRCA) of the three subspecies is positioned by using *F*. *novicida* and *F*. *hispaniensis* to root the tree (**S2 Table**). The MRCA is located on the longest branch, which separates *holarctica* from *mediasiatica* and *tularensis*. The topology of the tree is in full agreement with previous reports [6, 31, 34–36]. The distance from the MRCA to the tips is relatively homogeneous within each subspecies. Within *holarctica*, this distance goes from 2000 in lineage B.V (currently called B.16) up to 2400 in lineage B.IV (currently called B.6). The distances within *mediasiatica* are approximately 2400 with little variations. The distances within *tularensis* range from approximately 1130 in A.II up to 1290 in A.I, significantly shorter than in the other two subspecies.

### Determination of subspecies by biochemical properties

In order to confirm the subspecies assignment, we examined the biochemical properties of the Altaic strains. Subspecies *tularensis* and *mediasiatica* may be distinguished from subsp. *holarctica* by their capacity to ferment glycerol and their possession of the enzyme citrulline ureidase [37, 38]. The main biochemical difference between subspecies *tularensis* and *mediasiatica* is beta-lactamase activity: subsp. *mediasiatica* does not exhibit this activity [39]. We found that all Altaic strains were able to ferment glycerol and showed citrulline ureidase but not beta-lactamase activity. Therefore, we concluded that these strains belonged to subsp. *mediasiatica*.

### Virulence determination

**Fig 7:**
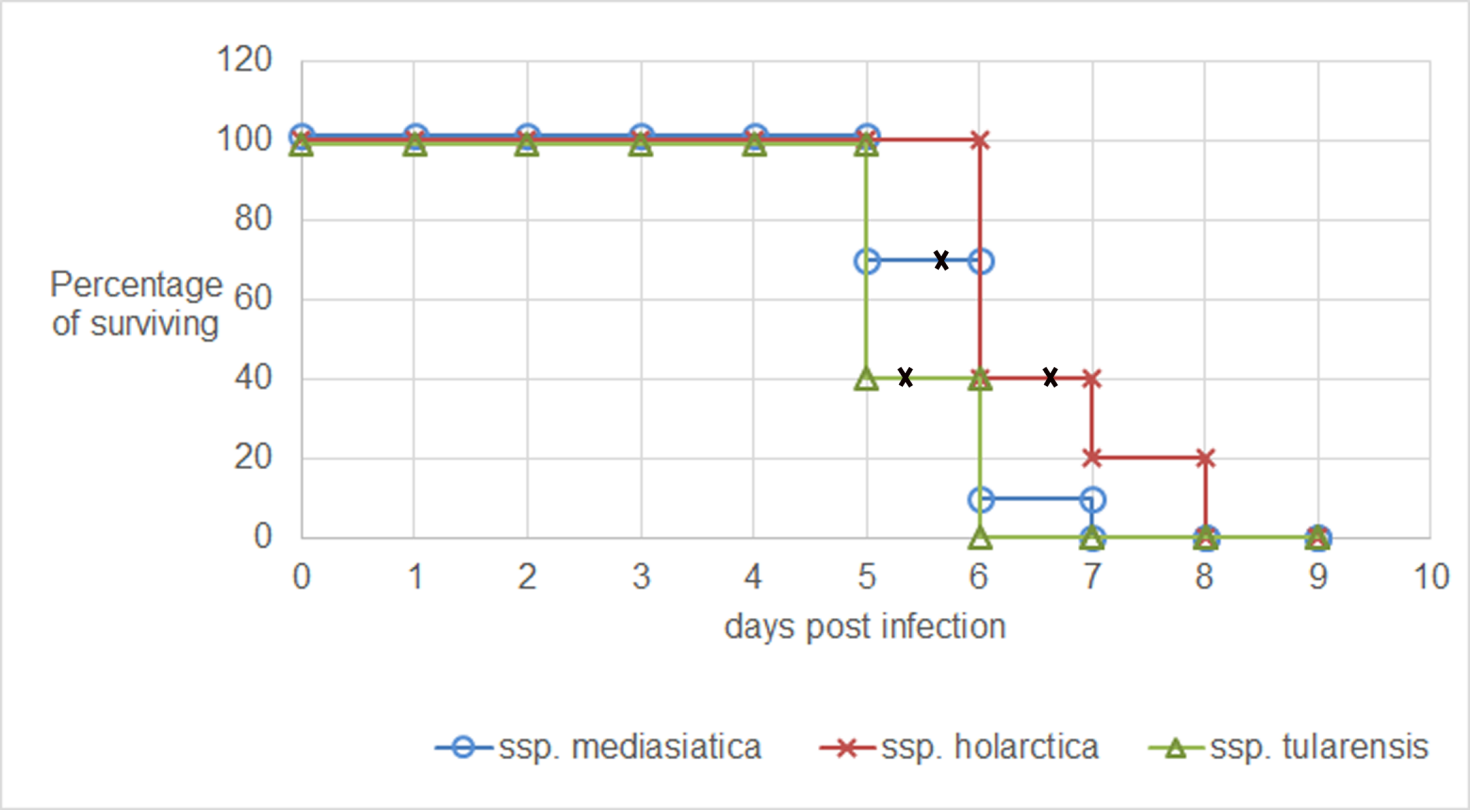
Survival curves of naive mice following challenge with *F*. *tularensis* subspecies *tularensis*, *holarctica*, or *mediasiatica*. Step-down survival curves showing the percentage of mice surviving over 10 days post infection are presented. Data is shown for subsp. *mediasiatica* (strains A678, A823, A554, and 120), subsp. *holarctica* (strains A1045 and 503), and subsp. *tularensis* (SCHU S4). The mean time to death is denoted as a bold black cross.

For a determination of the virulence of the Altaic strains, we used a mouse model. BALB/c mice died after subcutaneous injection of Altai-origin *F*. *tularensis* (< 10 bacterial cells per mice) (**S3 Table** in supplementary materials). **S3 Table** shows that all mice died between the fifth and eighth day post infection. In **Fig 7,** the survival curves of mice infected with the minimum dose are presented.

**Fig 8:**
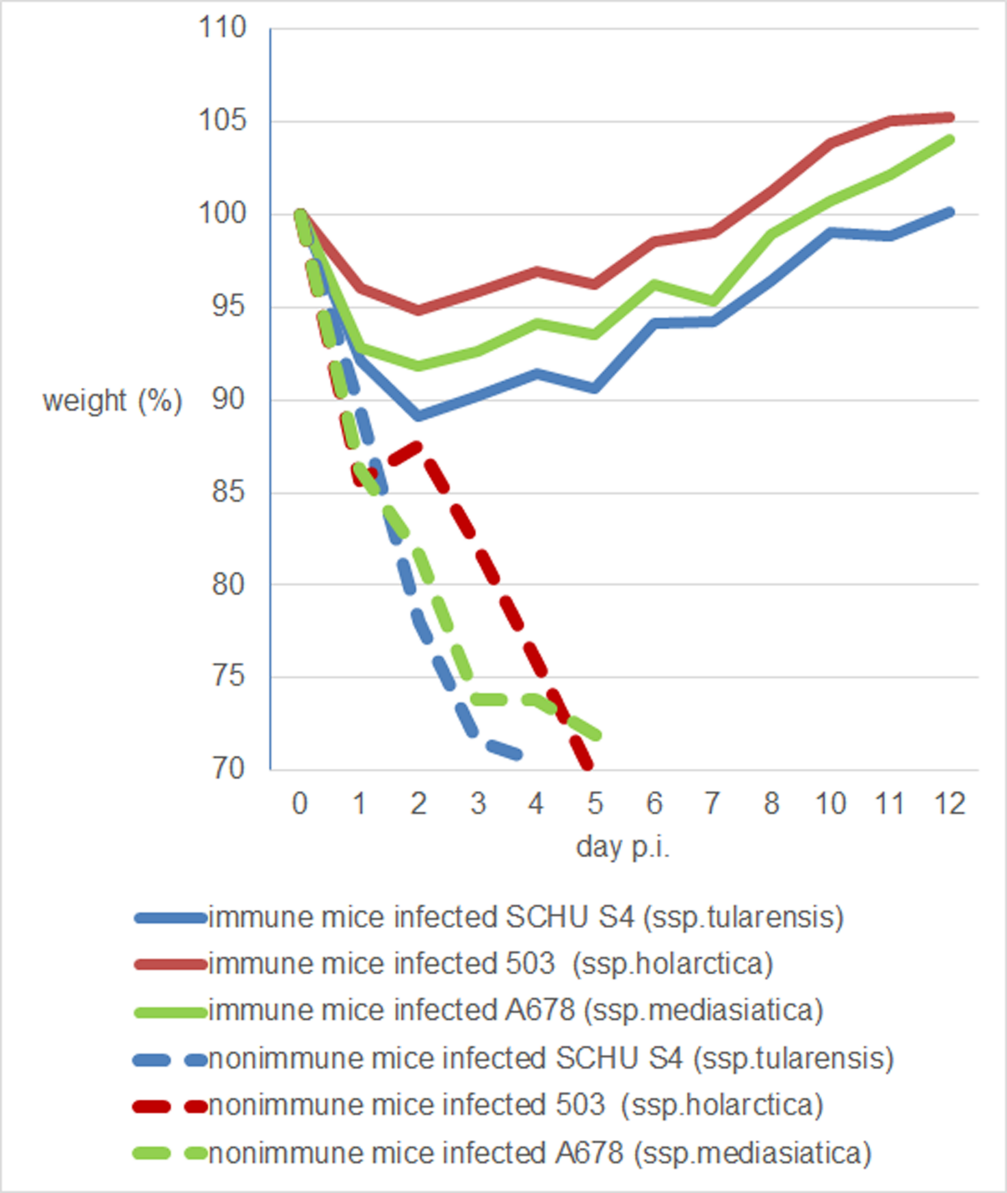
Weight loss (%) of immunized BALB/c mice after challenge with highly virulent strains of *F*. *tularensis*. Average weight loss through time in vaccinated mice infected with pathogenic strains of the different subspecies. Red line, weight loss in mice challenged with *holarctica* strain 503; Blue line, weight loss in mice challenged with *tularensis* strain SCHU S4; Green line, weight loss in mice challenged with *mediasiatica* strain A678. Solid line, immune mice; dashed line, non-immune mice (negative controls). All nonimmune mice died by 5 days postinfection. Body weight loss was calculated as the percentage of initial body weight in four experiments.

The average time to death of mice infected with the minimum dose (about 5–10 CFU per mice, see **S3 Table**) was 5.8±0.6 days for subsp. *mediasiatica*, 6.6± 0.8 days for subsp. *holarctica*, and 5.4±0.5 days for subsp. *tularensis* **(Fig 7)**. Student’s *t*-testing showed no statistically significant differences between the groups (p > 0.05). Therefore, the virulence of the Altaic strains was similar to the virulence of the other subspecies investigated. Because the high pathogenicity of *F*. *tularensis* in this animal model did not allow determining differences in the virulence of different subspecies, we decided to use vaccinated mice, which are significantly more resistant to *F*. *tularensis*. Mice were immunized subcutaneously with the *F*. *tularensis* 15 NIIEG vaccine strain (20 CFU per mouse) commonly used in Russia and some post-Soviet countries (see **S1 Table**). After 21 days, the mice were infected subcutaneously with strains A678 (*mediasiatica*), SCHU S4 (*tularensis*), and 503 (*holarctica*) (1000 CFU per mouse). This experiment was conducted with four independent replicates. Virulence was evaluated by determining the mortality rate and disease severity, which was estimated based on the loss in body weight of infected mice. The weight loss of vaccinated mice after infection with pathogenic strains is shown in **S4 Table**. The average weight loss (%) through time is shown in **Fig 8**.

The mortality rate in mice challenged with *mediasiatica* strain A678 was 8% (2/24) and in those challenged with *tularensis* strain SCHU S4 was 12.5% (3/24). All mice challenged with *holarctica* strain 503 survived. Maximum weight loss was registered between days 5 and 8 after infection. Weight loss caused by challenge with strain A678 was intermediate between the weight loss caused by challenge with strains SCHU S4 and 503 **(Fig 7)**. On the fifth day postinfection, weight loss was 9.4±1% after infection with strain SCHU S4, 6.5±1.3% with strain A678, and 3.7±1.2% with strain 503 (p<0.05). Thus, we found a statistically significant difference in virulence among the subspecies tested. Virulence of subsp. *mediasiatica* strain A678 is intermediate between that of subspp. *tularensis* strain SCHU S4 and *holarctica* strain 503.

## Discussion

The isolation of a number of subsp. *mediasiatica* strains in the Altai territory suggests the existence of a natural focus of *F*. *tularensis* subsp. *mediasiatica* circulation. This focus is located more than 1500 km eastward from previously identified foci in Kazakhstan and Turkmenistan.

The MLVA data clearly indicates that the Altai population of *F*. *tularensis* subsp. *mediasiatica* is genetically distinct and likely endemic to the Altai region. Thus, currently known strains of subsp. *mediasiatica* can be divided into three genetic groups, which we propose to call M.I, M.II and M.III. Our data adds to the present knowledge of the intra-specific structure of *F*. *tularensis*. To date, intra-subspecific clustering were proposed only for subsp. *tularensis* and *holarctica*. Based on MLVA [25], Johansson et al. divided subsp. *tularensis* into two subgroups (A.I and A.II) and subsp. *holarctica* into five subgroups (B.I–B.V). We propose dividing subsp. *mediasiatica* into three groups, M.I, M.II and M.III based on both genotype (MLVA-profile) and geographical location. M.III is defined by a single strain, 60B-57 from Karakalpastan in Western Uzbekistan, no strain from Turkmenistan could be investigated here.

Thus, we have found that Altai has genetically and geographically distinct population of *F*. *tularensis* subsp. *mediasiatica* circulating, and that the distribution of this subspecies is much broader than previously believed. We believe that the lack of earlier reports results mainly from the extremely low human population in Siberia and inaccessibility of some of its regions (mountainous Altai in particular).

These extreme conditions greatly complicate both the identification of pathogens and diagnosis of the disease in this environment. To date, there have been no reported cases of tularemia in humans caused by the subsp. *mediasiatica* in Altai. Nevertheless, according to the Chief State Sanitary Doctor of the Altai region (http://7law.info/altajsky/act2i/u298.htm), cases of tularemia in humans are identified in this region almost every year. Importantly, when cases of human tularemia are detected, medical and epidemic services usually do not diagnose the subspecies of pathogen—they only identify *F*. *tularensis*. Difficulties in transport of the strains to specialized research laboratories make further investigation difficult. Thus, on site research is needed to answer the question of whether tularemia is caused by subsp. *mediasiatica* or subsp. *holarctica*. To date, we have conducted focused research on ticks, rodents, soil, and water samples, but not on clinical samples. After the first detection of *F*. *tularensis* subsp. *mediasiatica* in the Altai region in 2013 [24], an intensive search for this microorganism, carried out by the Altai anti-plague station, allowed us to identify 15 strains of this subspecies in 2013–2014 described in this manuscript and six strains (which were not included in this report) in 2015. We hope that the simple genotyping assays proposed here will help develop procedures that can be applied on site at low cost on clinical as well as environmental samples. MLVA is especially interesting as compared to typing of a selection of SNPs because it is an unbiased approach, which will identify a previously unknown clade. The online *Francisella tularensis* database hosted on the Microbes Genotyping web site provides an easily accessible depository of reference data for comparisons.

Whole genome sequencing will help investigate more precisely the population structure of the M.II group, as illustrated by the investigation of other *F*. *tularensis* foci [40] and the evolutionary relationships between subsp. *mediasiatica*, *holarctica* and *tularensis*. Wang et al [41] recently described the presence of all basal *holarctica* lineages in Western China. The present description of *mediasiatica* in Altai represents one additional step pointing to similarities between the phylogeography of *F*. *tularensis* and *Yersinia pestis*. The most basal *Y*. *pestis* lineages showing no virulence in humans are also found in Altai, at the junction between China, Mongolia, Russia and Kazakhstan [42–44]. The two species share similar ecological behaviors and vectors, it will be interesting to investigate to which extend their phylogeography is coincident.

## Conclusion

We showed that the geographic distribution of *F*. *tularensis* subsp. *mediasiatica* is much wider than previously believed, including not only Central Asia but also Southern Siberia and the Altai region of Russia. Because strains of Altaic origin are genetically distinct from strains of Central Asian origin, we propose the division of subsp. *mediasiatica* into two intra-subspecies phylogeographic groups called M.I (Central Asian origin) and M.II (Altaic origin). In addition, we showed that the virulence of subsp. *mediasiatica* in a vaccinated mouse model is intermediate between that of subsp. *tularensis* and subsp. *holarctica*.

## Supporting information

S1 Appendix 2% agarose- electrophoresis of Ft-M3 PCR-products of 8 *F*. *tularensis* strains. M1-100 bp plus ladder, M2- 20 bp ladder; the 500 bp and 1 kb bands are slightly more intense 1—A99 subsp. mediasiatica (75 repeats, 882 bp), 2- A823 subsp. mediasiatica (63 repeats, 774 bp), 3—A116 subsp. mediasiatica (52 repeats, 675 bp), 4–120 subsp. mediasiatica (19 repeats, 378 bp), 5—A373 subsp. mediasiatica (53 repeats, 684 bp), 6—I346 subsp holarctica (11 repeats, 306 bp), 7—A139 subsp. mediasiatica (26 repeats, 441 bp), 8–319/358 subsp holarctica (24 repeats, 423 bp). **Interface of PhotoCaptMw with the calculation of the size of amplicons.**
(PDF)

S1 Table The strains of F. tularensis used in this study and MLVA15 profiles. (XLSX)

S2 Table (XLSX)

S3 Table BALB/c mice death after infection with F. tularensis strains of different subspecies. (XLSX)

S4 Table Body weight (g) of preliminarily vaccinated mice after infection with pathogenic strains A678, SCHUS4, and 503. (XLSX)

## References

[1] Dennis DT, Inglesby TV, Henderson DA, Bartlett JG, Ascher MS, Eitzen E, et al Tularemia as a biological weapon: medical and public health management. JAMA. 2001;285(21):2763–73. doi: 10.1001/jama.285.21.2763 11386933 10.1001/jama.285.21.2763

[2] Keim P, Johansson A, Wagner DM. Molecular epidemiology, evolution, and ecology of *Francisella*. Ann N Y Acad Sci. 2007;1105:30–66. doi: 10.1196/annals.1409.011 . 17435120 10.1196/annals.1409.011

[3] Telford SR 3rd, Goethert HK. Toward an understanding of the perpetuation of the agent of tularemia. Front Microbiol. 2010;1:150 doi: 10.3389/fmicb.2010.00150 21687803 10.3389/fmicb.2010.00150 PMC3109306

[4] Kingry LC, Petersen JM. Comparative review of *Francisella tularensis* and *Francisella novicida*. Front Cell Infect Microbiol. 2014;4:35 doi: 10.3389/fcimb.2014.00035 Epub 2014/03/25. 24660164 10.3389/fcimb.2014.00035 PMC3952080

[5] Gürcan Ş. Epidemiology of tularemia. Balkan Med J. 2014;31(1):3–10. doi: 10.5152/balkanmedj.2014.13117 25207161 10.5152/balkanmedj.2014.13117 PMC4115998

[6] Kiliç S, Birdsell DN, Karagöz A, Çelebi B, Bakkaloglu Z, Arikan M, et al Water as Source of *Francisella tularensis* Infection in Humans, Turkey. Emerging infectious diseases. 2015;21(12):2213–6. doi: 10.3201/eid2112.150634 26583383 10.3201/eid2112.150634 PMC4672436

[7] Champion MD, Zeng Q, Nix EB, Nano FE, Keim P, Kodira CD, et al Comparative genomic characterization of *Francisella tularensis* strains belonging to low and high virulence subspecies. PLoS Pathog. 2009;5(5):e1000459 doi: 10.1371/journal.ppat.1000459 19478886 10.1371/journal.ppat.1000459 PMC2682660

[8] Tärnvik A, Berglund L. Tularaemia. Eur Respir J. 2003;21(2):361–73. . 12608453 10.1183/09031936.03.00088903

[9] McLendon MK, Apicella MA, Allen LA. *Francisella tularensis*: taxonomy, genetics, and Immunopathogenesis of a potential agent of biowarfare. Annu Rev Microbiol. 2006; 60:167–85. doi: 10.1146/annurev.micro.60.080805.142126 16704343 10.1146/annurev.micro.60.080805.142126 PMC1945232

[10] Kugeler KJ, Mead PS, Janusz AM, Staples JE, Kubota KA, Chalcraft LG, et al Molecular Epidemiology of *Francisella tularensis* in the United States. Clin Infect Dis. 2009;48(7):863–70. doi: 10.1086/597261. 19245342 10.1086/597261

[11] Leelaporn A, Yongyod S, Limsrivanichakorn S, Yungyuen T, Kiratisin P. *Francisella novicida* bacteremia, Thailand. Emerg Infect Dis. 2008;14(12):1935–7. doi: 10.3201/eid1412.080435 19046526 10.3201/eid1412.080435 PMC2634620

[12] Whipp MJ, Davis JM, Lum G, de Boer J, Zhou Y, Bearden SW, et al Characterization of a novicida-like subspecies of *Francisella tularensis* isolated in Australia. J Med Microbiol. 2003;52(Pt 9):839–42. doi: 10.1099/jmm.0.05245-0 12909664 10.1099/jmm.0.05245-0

[13] Huber B, Escudero R, Busse HJ, Seibold E, Scholz HC, Anda P, et al Description of *Francisella hispaniensis* sp. nov., isolated from human blood, reclassification of *Francisella novicida* (Larson et al. 1955) Olsufiev et al. 1959 as *Francisella tularensis* subsp. *novicida* comb. nov. and emended description of the genus *Francisella*. Int J Syst Evol Microbiol. 2010;60(Pt 8):1887–96. doi: 10.1099/ijs.0.015941-0 19783615 10.1099/ijs.0.015941-0

[14] Johansson A, Celli J, Conlan W, Elkins KL, Forsman M, Keim PS, et al Objections to the transfer of *Francisella novicida* to the subspecies rank of *Francisella tularensis*. Int J Syst Evol Microbiol. 2010;60 (Pt 8):1717–8; doi: 10.1099/ijs.0.022830-0 20688748 10.1099/ijs.0.022830-0 PMC7442299

[15] Pandya GA, Holmes MH, Petersen JM, Pradhan S, Karamycheva SA, Wolcott MJ, et al Whole genome single nucleotide polymorphism based phylogeny of *Francisella tularensis* and its application to the development of a strain typing assay. BMC Microbiol. 2009;9:213 doi: 10.1186/1471-2180-9-213 19811647 10.1186/1471-2180-9-213 PMC2767358

[16] Challacombe JF, Petersen JM, Gallegos-Graves V, Hodge D, Pillai S, Kuske CR. Whole-Genome Relationships among *Francisella* Bacteria of Diverse Origins Define New Species and Provide Specific Regions for Detection. Appl Environ Microbiol. 2017;83(3). doi: 10.1128/AEM.02589-16 10.1128/AEM.02589-16 PMC5244304 27881415

[17] Birdsell DN, Stewart T, Vogler AJ, Lawaczeck E, Diggs A, Sylvester TL, et al *Francisella tularensis* subsp. *novicida* isolated from a human in Arizona. BMC Res Notes. 2009;2:223 doi: 10.1186/1756-0500-2-223 19895698 10.1186/1756-0500-2-223 PMC2780447

[18] Clarridge JE, 3rd, Raich TJ, Sjösted A, Sandström G, Darouiche RO, Shawar RM, et al Characterization of two unusual clinically significant *Francisella* strains. J Clin Microbiol. 1996;34(8):1995–2000. 8818897 10.1128/jcm.34.8.1995-2000.1996 PMC229169

[19] Hollis DG, Weaver RE, Steigerwalt AG, Wenger JD, Moss CW, Brenner DJ. *Francisella philomiragia* comb. nov. (formerly Yersinia philomiragia) and *Francisella tularensis* biogroup *novicida* (formerly *Francisella novicida*) associated with human disease. J Clin Microbiol. 1989;27(7):1601–8. 2671019 10.1128/jcm.27.7.1601-1608.1989 PMC267622

[20] Olsufjev N.G., Meshcheryakova I. S. Subspecific taxonomy of Francisella tularensis McCoy and Chapin 1912. Int. J. Syst. Bacteriol. 1983, vol. 33, pp. 872–874. doi: 10.1099/00207713-33-4-87221

[21] Aikimbaev MA. Taxonomy of the genus *Francisella*. Rep Acad Sci Kaz SSR Ser Biol. 1966;5:42–4.

[22] Broekhuijsen M, Larsson P, Johansson A, Byström M, Eriksson U, Larsson E, et al Genome-wide DNA microarray analysis of *Francisella tularensis* strains demonstrates extensive genetic conservation within the species but identifies regions that are unique to the highly virulent *F*. *tularensis* subsp. *tularensis*. J Clin Microbiol. 2003;41(7):2924–31. doi: 10.1128/JCM.41.7.2924-2931.2003 12843022 10.1128/JCM.41.7.2924-2931.2003 PMC165330

[23] Olsufjev N.G., Meshcheryakova I. S. Subspecific taxonomy of Francisella tularensis McCoy and Chapin 1912. Int. J. Syst. Bacteriol. 1983, 33, pp. 872–874. doi: 10.1099/00207713-33-4-872.

[24] Mokrievich AN, Timofeev VS, Kudryavtseva TY, Ulanova GI, Karbysheva SB, Mironova RI, et al Isolation of Central Asian subspecies of tularemia agent in the Altai territory. Prob Espec Dang Infec. 2013;115:66–9.

[25] Johansson A, Farlow J, Larsson P, Dukerich M, Chambers E, Byström M, et al Worldwide genetic relationships among *Francisella tularensis* isolates determined by multiple-locus variable-number tandem repeat analysis. J Bacteriol. 2004;186(17):5808–18. doi: 10.1128/JB.186.17.5808-5818.2004 15317786 10.1128/JB.186.17.5808-5818.2004 PMC516809

[26] Rodionova IV. [Differentiation of geographic races of *Francisella tularensis* on the basis of citrulline ureidase activity]. Lab Delo. 1970;1:42–3. Epub 1970/01/01. . 4192178

[27] Gaitonde MK. A spectrophotometric method for the direct determination of cysteine in the presence of other naturally occurring amino acids. Biochem J. 1967;104(2):627–33. PubMed Central PMCID: PMC1270629. 6048802 10.1042/bj1040627 PMC1270629

[28] Qin A, Zhang Y, Clark ME, Rabideau MM, Millan Barea LR, Mann BJ. FipB, an essential virulence factor of *Francisella tularensis* subsp. *tularensis*, has dual roles in disulfide bond formation. J Bacteriol. 2014;196(20):3571–81. doi: 10.1128/JB.01359-13 25092026 10.1128/JB.01359-13 PMC4187702

[29] O'Sullivan MV, Sintchenko V, Gilbert GL. Software for selecting the most informative sets of genomic loci for multi-target microbial typing. BMC Bioinformatics. 2013;14:148 doi: 10.1186/1471-2105-14-148 23635100 10.1186/1471-2105-14-148 PMC3660239

[30] Gürcan S, Karabay O, Karadenizli A, Karagol C, Kantardjiev T, Ivanov IN. Characteristics of the Turkish isolates of *Francisella tularensis*. Jpn J Infect Dis. 2008;61(3):223–5. . 18503176

[31] Vogler AJ, Birdsell D, Price LB, Bowers JR, Beckstrom-Sternberg SM, Auerbach RK, et al Phylogeography of *Francisella tularensis*: global expansion of a highly fit clone. J Bacteriol. 2009;191(8):2474–84. doi: 10.1128/JB.01786-08 19251856 10.1128/JB.01786-08 PMC2668398

[32] Williams JG, Kubelik AR, Livak KJ, Rafalski JA, Tingey SV. DNA polymorphisms amplified by arbitrary primers are useful as genetic markers. Nucleic Acids Res. 1990;18(22):6531–5. PubMed Central PMCID: PMC332606. 1979162 10.1093/nar/18.22.6531 PMC332606

[33] Larsson P, Oyston PC, Chain P, Chu MC, Duffield M, Fuxelius HH, et al The complete genome sequence of *Francisella tularensis*, the causative agent of tularemia. Nat Genet. 2005;37(2):153–9. doi: 10.1038/ng1499 15640799 10.1038/ng1499

[34] Sjodin A, Svensson K, Ohrman C, Ahlinder J, Lindgren P, Duodu S, et al Genome characterisation of the genus Francisella reveals insight into similar evolutionary paths in pathogens of mammals and fish. BMC Genomics. 2012;13:268 doi: 10.1186/1471-2164-13-268 22727144 10.1186/1471-2164-13-268 PMC3485624

[35] Karlsson E, Svensson K, Lindgren P, Byström M, Sjödin A, Forsman M, et al The phylogeographic pattern of *Francisella tularensis* in Sweden indicates a Scandinavian origin of Eurosiberian tularaemia. Environ Microbiol. 2013;15(2):634–45. doi: 10.1111/1462-2920.12052 23253075 10.1111/1462-2920.12052

[36] Gyuranecz M, Birdsell DN, Splettstoesser W, Seibold E, Beckstrom-Sternberg SM, Makrai L, et al Phylogeography of *Francisella tularensis* subsp. *holarctica*, Europe. Emerging infectious diseases. 2012;18(2):290–3. doi: 10.3201/eid1802.111305 22305204 10.3201/eid1802.111305 PMC3310461

[37] Sandström G, Sjöstedt A, Forsman M, Pavlovich NV, Mishankin BN. Characterization and classification of strains of *Francisella tularensis* isolated in the central Asian focus of the Soviet Union and in Japan. J Clin Microbiol. 1992;30(1):172–5. PubMed Central PMCID: PMC265015. 1370846 10.1128/jcm.30.1.172-175.1992 PMC265015

[38] Sjöstedt A. *Francisella* The Proteobacteria, part B In: Brenner DJ, Krieg NR, Staley JT, editors. Bergey's manual of systematic bacteriology. New York: Springer-Verlag; 2005 p. 200–10.

[39] Sjöstedt A. Family XVII, Francisellaceae. Genus I, *Francisella* In: Garrity GM, editor. Bergey's manual of systematic bacteriology. 2 2nd ed New York: Springer-Verlag; 2003 p. 111–3.

[40] Myrtennäs K, Marinov K, Johansson A, Niemcewicz M, Karlsson E, Byström M, et al Introduction and persistence of tularemia in Bulgaria. Infect Ecol Epidemiol. 2016;6:32838 doi: 10.3402/iee.v6.32838 27790972 10.3402/iee.v6.32838 PMC5084392

[41] Wang Y, Peng Y, Hai R, Xia L, Li H, Zhang Z, et al Diversity of *Francisella tularensis* subsp. *holarctica* lineages, China. Emerg Infect Dis. 2014;20(7):1191–4. doi: 10.3201/eid2007.130931 24963721 10.3201/eid2007.130931 PMC4073844

[42] Riehm JM, Vergnaud G, Kiefer D, Damdindorj T, Dashdavaa O, Khurelsukh T, et al *Yersinia pestis* lineages in Mongolia. PLoS One. 2012;7(2):e30624 Epub 2012/03/01. doi: 10.1371/journal.pone.0030624 22363455 10.1371/journal.pone.0030624 PMC3281858

[43] Li Y, Cui Y, Hauck Y, Platonov ME, Dai E, Song Y, et al Genotyping and phylogenetic analysis of *Yersinia pestis* by MLVA: insights into the worldwide expansion of Central Asia plague foci. PLoS One. 2009;4(6):e6000 doi: 10.1371/journal.pone.0006000 19543392 10.1371/journal.pone.0006000 PMC2694983

[44] Cui Y, Yu C, Yan Y, Li D, Li Y, Jombart T, et al Historical variations in mutation rate in an epidemic pathogen, *Yersinia pestis*. Proc Natl Acad Sci U S A. 2013;110(2):577–82. doi: 10.1073/pnas.1205750110 23271803 10.1073/pnas.1205750110 PMC3545753

