# Supplementary material for: Russian isolates enlarge the known geographic diversity of Francisella tularensis subsp. mediasiatica
Source: PLoS One. 2017 Sep 5;12(9):e0183714. doi: 10.1371/journal.pone.0183714 (PMC5584958; doi:10.1371/journal.pone.0183714)
Supplement: S1 Appendix — M1-100 bp plus ladder, M2- 20 bp ladder; the 500 bp and 1 kb bands are slightly more intense 1—A99 subsp. mediasiatica (75 repeats, 882 bp), 2- A823 subsp. mediasiatica (63 repeats, 774 bp), 3—A116 subsp. mediasiatica (52 repeats, 675 bp), 4–120 subsp. mediasiatica (19 repeats, 378 bp), 5—A373 subsp. mediasiatica (53 repeats, 684 bp), 6—I346 subsp holarctica (11 repeats, 306 bp), 7—A139 subsp. mediasiatica (26 repeats, 441 bp), 8–319/358 subsp holarctica (24 repeats, 423 bp). Interface of PhotoCaptMw with the calculation of the size of amplicons. (PDF) [file pone.0183714.s001.pdf]

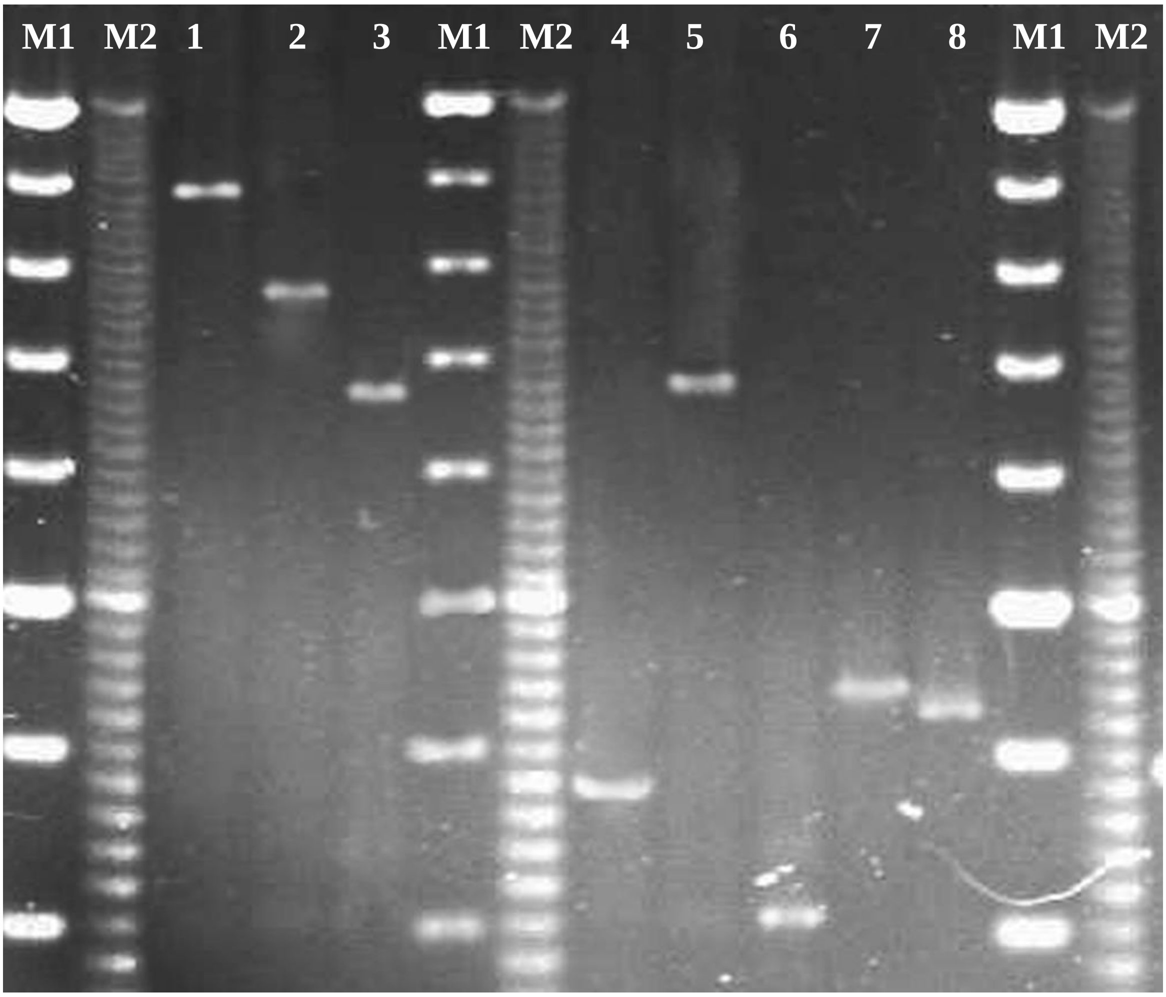

2% agarose- electrophoresis of Ft-M3 PCR-products of 8 *F. tularensis* strains

M1-100 bp plus ladder, M2- 20 bp ladder; the 500 bp and 1 kb bands are slightly more intense 1 - A99 subsp. *mediasiatica* (75 repeats, 882 bp), 2- A823 subsp. *mediasiatica* (63 repeats, 774 bp), 3 - A116 subsp. *mediasiatica* (52 repeats, 675 bp), 4 - 120 subsp. *mediasiatica* (19 repeats, 378 bp), 5 - A373 subsp. *mediasiatica* (53 repeats, 684 bp), 6 - I346 subsp *holarctica* (11 repeats, 306 bp), 7 - A139 subsp. *mediasiatica* (26 repeats, 441 bp), 8 - 319/358 subsp *holarctica* (24 repeats, 423 bp).

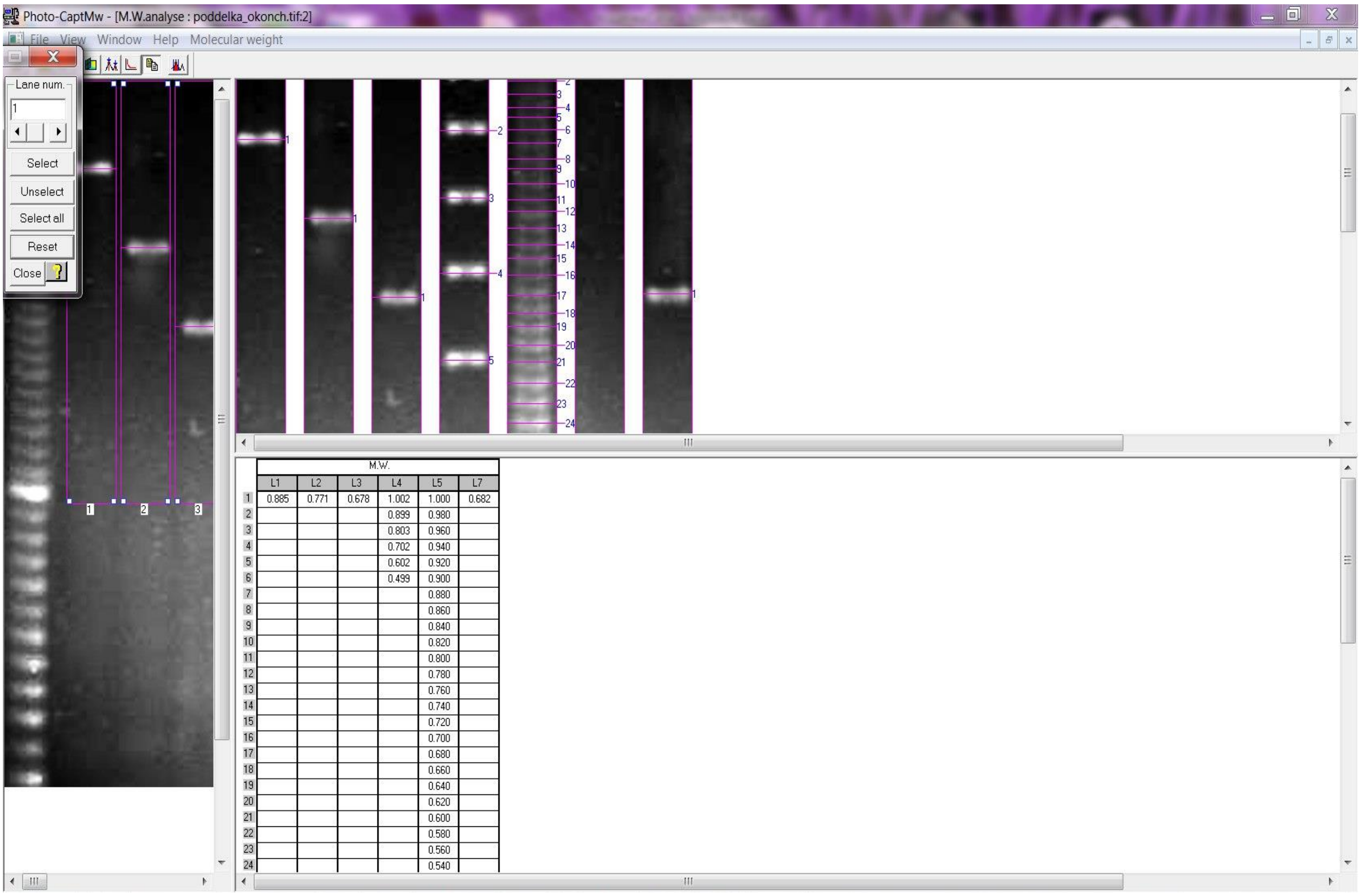

Interface of PhotoCaptMw with the calculation of the size of amplicons
